# Limitations of current *in vitro* models for testing the clinical potential of epigenetic inhibitors for treatment of pediatric ependymoma

**DOI:** 10.18632/oncotarget.26370

**Published:** 2018-11-23

**Authors:** Hazel Anne Rogers, Rebecca Chapman, Holly Kings, Julie Allard, Jodie Barron-Hastings, Kristian W. Pajtler, Martin Sill, Stefan Pfister, Richard Guy Grundy

**Affiliations:** ^1^ Children's Brain Tumour Research Centre, School of Medicine, University of Nottingham, Nottingham, UK; ^2^ Hopp Children's Cancer Centre at the NCT (KiTZ), Heidelberg, Germany; ^3^ German Cancer Research Centre (DKFZ) and German Cancer Consortium (DKTK), Heidelberg, Germany; ^4^ Department of Haematology and Oncology, University Hospital, Heidelberg, Germany

**Keywords:** ependymoma, brain tumor, epigenetics, pediatric

## Abstract

**Background:**

Epigenetic modifications have been shown to play an important role in the classification and pathogenesis of the pediatric brain tumor ependymoma, suggesting they are a potential therapeutic target.

**Results:**

Agents targeting epigenetic modifications inhibited the growth and induced the death of ependymoma cells with variable efficiency. However, this was often not at clinically achievable doses. Additionally, DNA methylation profiling revealed a lack of similarity to primary ependymomas suggesting alterations were induced during culture. Toxicity to fetal neural stem cells was also seen at similar drug concentrations

**Conclusions:**

Agents targeting epigenetic modifications were able to inhibit the growth and induced the death of ependymoma cells grown *in vitro*. However, many agents were only active at high doses, outside clinical ranges, and also resulted in toxicity to normal brain cells. The lack of similarity in DNA methylation profiles between cultured cells and primary ependymomas questions the validity of using *in vitro* cultured cells for pre-clinical analysis of agents targeting epigenetic mechanisms and suggests further investigation using models that are more appropriate should be undertaken before agents are taken forward for clinical testing.

**Materials and Methods:**

The effects of agents targeting epigenetic modifications on the growth and death of a panel of ependymoma cell lines was investigated, as well as toxicity to normal fetal neural stem cells. The ependymoma cell lines were characterized using DNA methylation profiling.

## INTRODUCTION

Ependymoma is the second most common malignant pediatric tumor of the CNS, accounting for approximately 10% of all brain tumors in children [1]. Prognosis remains relatively poor and little progress has been made in recent decades to develop new treatments.

Epigenetic modifications can be defined as stable and heritable changes in gene expression resulting from modifications to the DNA molecule without alterations in the DNA sequence. Epigenetic alterations play a role in the development and progression of many cancers. The most common types of modification altered include DNA methylation, histone acetylation and histone methylation [2–5].

In cancer, increased DNA methylation at promoters of tumor suppressor genes, resulting in their silencing, is often seen. Therefore, de-methylating agents have been investigated for therapeutic use. One of the most advanced is decitabine, which targets DNA methyltransferase enzymes. It has been approved by the Food and Drug Administration (FDA) for the treatment of myelodysplastic syndrome as well as being investigated in clinical trials for other cancers [6, 7].

Histone acetylation plays a key role in the regulation of gene expression and levels are often altered in cancer. Histone de-acetylase inhibitors (HDACi) are the most clinically advanced agents targeting epigenetic modifications. A number of agents targeting this modification are FDA approved, including vorinostat, for the treatment of cutaneous T-cell lymphoma, and panobinostat, for the treatment of multiple myeloma [8, 9].

Histone methylation also plays an important role in cancer development and progression [10]. In particular, the repressive tri-methylation mark on lysine 27 of histone H3 (H3K27) has been shown to be important for pediatric brain tumors including ependymoma [11, 12]. H3K27 is methylated by the polycomb repressor complex 2 (PRC2). Enhancer of zeste homolog 2 (EZH2), the catalytic subunit of PRC2, is commonly mutated or overexpressed in cancer [13]. Inhibitors targeting EZH2 have been developed including GSK343 and EPZ-6438 [14–16].

Recent studies have highlighted the importance of epigenetic modifications in ependymoma [11, 17–19]. DNA methylation profiling has demonstrated this epigenetic mark can define ependymoma sub-groups based on demographic and clinical features, including age at diagnosis and tumor location [11, 17]. For pediatric patients, DNA methylation defined two posterior fossa (PF) groups, EPN_PFA and EPN_PFB, and two supratentorial (ST) groups, EPN_RELPOS and EPN_YAP1. EPN_PFA tumors were shown to occur in young children and displayed a hyper-methylated phenotype. EPN_PFB tumors occurred in older children and displayed a hypo-methylated phenotype [11]. EPN_RELPOS and EPN_YAP1 ependymomas were characterized by recently discovered fusion genes involving RELA and YAP1 respectively [20].

Initial pre-clinical evidence supports the potential therapeutic targeting of epigenetic modifications in ependymoma. Mack *et al* demonstrated decitabine and GSK343 inhibited the growth of primary ependymoma cultures *in vitro*. Studies also support the clinical potential of HDAC inhibitors in ependymoma [18, 21].

Pre-clinical analysis of agents targeting epigenetic modifications in ependymoma has so far been undertaken using a small set of *in vitro* cultured cells. However, the epigenetic profile of these models was not investigated. The clinical relevance of *in vitro* models has long been questioned and studies have shown that epigenetic modifications can alter during cell culture [22–24].

In this study, we have expanded the pre-clinical investigation of agents targeting DNA methylation, histone acetylation and H3K27 methylation using a panel of ependymoma cell lines. Alongside this, we used DNA methylation profiling to measure how closely the cultured cells retained their original profiles. We show that epigenetic agents inhibited the growth and induced the death of ependymoma cells with variable efficacy, but was often outside clinically achievable ranges. Importantly, DNA methylation profiling of the cultured ependymoma cells indicated their profiles were altered from that seen in primary ependymoma tumor tissue for the majority of the lines tested, questioning the validity of *in vitro* cultured cells for analysis of epigenetic agents.

## RESULTS

### The majority of ependymoma cell lines did not closely resemble defined molecular groups

Cultured cells derived from six ependymomas (3 ST, 3 PF) were used in the study. Analysis of C11orf95-RELA fusion status and DNA methylation profiling was used to characterize the cells.

**Figure 1:**
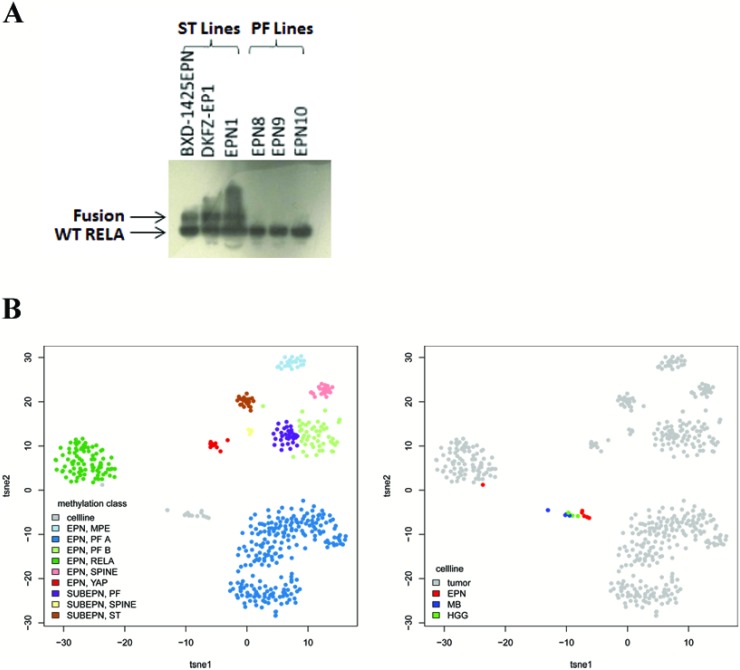
Cell line subgroup characterization C11orf95-RELA fusion status was determined using western blot for wild-type rela (**A**). A larger protein than the wild-type (WT), representing the fusion gene, was seen in all three ST cell lines but not in the PF cells. t-SNE dimension reduction demonstrated that cultured ependymoma cells mainly grouped together with cell lines derived from other brain tumors rather than primary ependymoma samples, with the exception of DKFZ-EP1, which clustered with EPN_RELPOS cells (**B**).

C11orf95-RELA fusion status was determined using western blot to wild-type RELA. Fusions (seen as larger proteins than wild-type rela) were detected in all cells derived from ST ependymomas (BXD-1425EPN, DKFZ-EP1, EPN1) but none of the PF cells (EPN8, EPN9, EPN10) (Figure 1A).

All cells underwent DNA methylation profiling using Infinium HumanMethylation450 BeadChip arrays (Illumina). Supervised class prediction by the previously published classifier [25] (www.molecularneuropathology.org) was used to assign cell line profiles to tumor subgroups. The only cell line that could be confidently assigned to an ependymoma molecular subgroup was DKFZ-EP1, which was classified as EPN_RELPOS. EPN10 was classified as EPN_PFA. However, this was with a low level of confidence. All other ependymoma cells were misclassified with very low confidence scores (Table 1). DNA methylation has been shown to alter during *in vitro* cell culture, which may explain these discrepancies [22–24]. Clustering of the cultured ependymoma cells alongside ependymoma tumors, plus cell lines from other brain tumor types, demonstrated that all the cultured cells formed one group, with the exception of the DKFZ-EP1 cell line which correctly grouped with the EPN_RELPOS ependymomas (Figure 1B). This suggested that *in vitro* culture induced DNA methylation changes in the cells, resulting in a common culture induced profile.

**Table 1:** Cell Line subgroup classification from DNA methylation profiles

Cell Line	Subgroup Prediction	Prediction Score^*^
BXD-1425EPN	CPC (high grade plexus tumor)	0.06
DKFZ-EP1	EPN_RELPOS	1.00
EPN1	MNG (meningioma)	0.07
EPN8	MNG (meningioma)	0.06
EPN9	MNG (meningioma)	0.07
EPN10	EPN_PFA	0.20

* 0 = 0% confidence, 1 = 100% confidence.

### Agents targeting epigenetic modifications inhibited the growth of ependymoma cells

**Figure 2:**
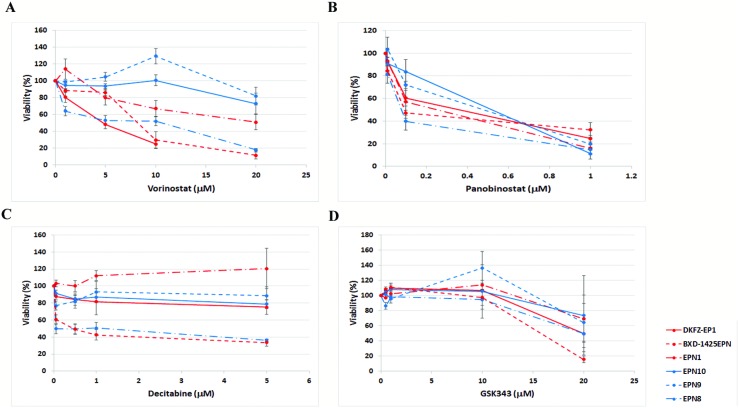
Effect of epigenetic agents on ependymoma cell line viability Epigenetic agents reduced the viability of ependymoma cells. Ependymoma cells were treated with vorinostat (**A**) and panobinostat (**B**), or vehicle control, for 72 hrs. Both agents reduced cell viability, relative to vehicle control, in the majority of ependymoma cells, including the DKFZ-EP1 cells. Ependymoma cells were incubated with decitabine (**C**) for 144 hrs. A decrease in viability was seen in only a minority of ependymoma cells, which did not include DKFZ-EP1 cells. Cells were incubated with GSK343 (**D**) for 120 hrs. GKS343 did not induce a decrease in viability in the majority of ependymoma cells tested. Cell viability was measured using a MTT assay.

To assess the clinical potential of agents targeting epigenetic modifications, ependymoma cells were incubated with selected agents and the effects on cell viability investigated (Figure 2). For each agent there was no clear difference between the more representative model, DKFZ-EP1, and the other ependymoma cells. There was also no clear difference between cells with (BXD-1425EPN, DKFZ-EP1, EPN1) or without (EPN8, EPN9, EPN10) the C11orf95-RELA fusion.

The HDAC inhibitors vorinostat and panobinostat were most effective, causing a significant decrease in viability in most cells tested (Figure 2A, 2B). IC50 concentrations for vorinostat ranged from 5 μM to greater than 20 μM, and panobinostat from 90–510 nM (Supplementary Table 1).

The de-methylating agent decitabine caused a significant reduction in viability in BXD-1425EPN and EPN8 cells (Figure 2C), which was only seen after 144 hrs. Only a modest effect on viability was seen in all other ependymoma cells tested. IC50 concentrations ranged from 50 nM to greater than 5 μM (Supplementary Table 1).

The EZH2 inhibitor GSK343 did not induce a significant reduction in viability for the majority of ependymoma cells tested (Figure 2D). The largest effect was seen for BXD-1425EPN and EPN8 cells. IC50 concentrations ranged from 16 μM to greater than 20 μM (Supplementary Table 1). The decrease in viability was only observed after 120 hrs.

### HDAC and EZH2 inhibition induced cell death

**Figure 3:**
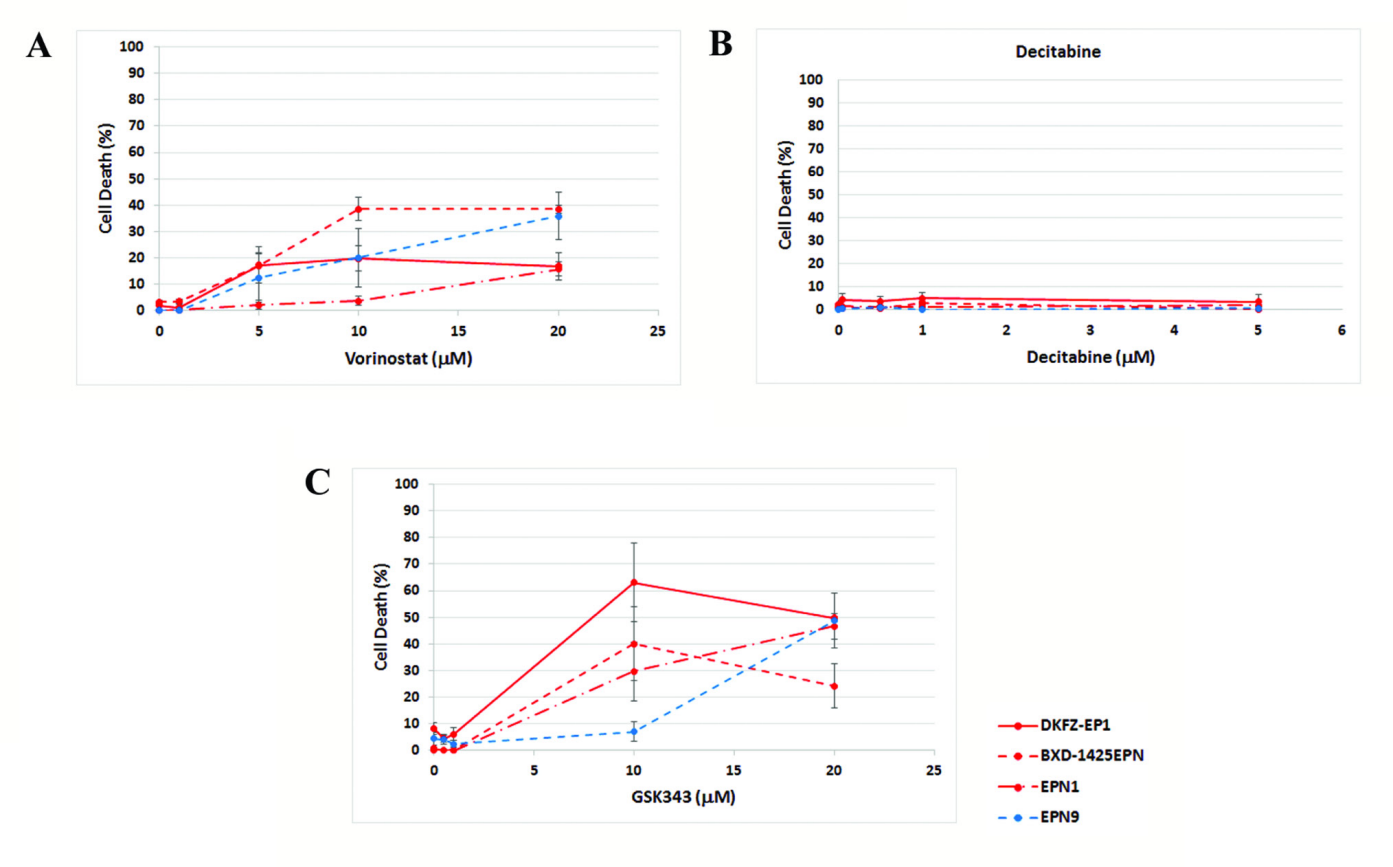
Effect of epigenetic agents on ependymoma cell death A LDH assay was used to measure whether cell death was induced by epigenetic agents. Vorinostat induced cell death in all ependymoma cells tested following 72 hrs treatment (**A**). Decitabine did not induce cell death in any ependymoma cells following treatment for 144 hrs (**B**). GSKS343 induced cell death following 120 hrs treatment (**C**).

Cell death was analyzed to determine whether the epigenetic agents induced a cytotoxic response. One drug was selected targeting each epigenetic mechanism. Cell death was measured using a LDH assay following vorinostat, decitabine and GSK343 treatment (Figure 3). There was again no clear difference in the level of cell death induced between the more representative DKFZ-EP1 cells and the other ependymoma cells, or between cells with or without the C11orf95-RELA fusion.

Vorinostat and GSK343 both induced cell death in ependymoma cells (Figure 3), suggesting this was the significant cause of decreased viability induced by both these agents. However, significant cell death was not induced by decitabine (Figure 3), suggesting a growth arrest was induced instead.

### Targeting multiple epigenetic mechanisms enhanced their inhibitory effects

The effect of targeting multiple epigenetic modifications was investigated to determine if a combinatorial approach enhanced their effects (Supplementary Figure 1). For combinations involving decitabine, cells were treated with decitabine alone before addition of the second drug. HDAC and EZH2 inhibitors can block the progression of G1 cells into S phase, which interferes with the action of decitabine [26, 27].

In general, no significant enhanced effects were seen between treatment with combinations or each drug alone (Supplementary Figure 1). No clear difference was seen in response to drug combinations between DKFZ-EP1 and other ependymoma cells tested.

Decitabine plus vorinostat showed some enhancement of effects in some cells tested. However, this was only seen at higher drug concentrations and was not seen in the DKFZ-EP1 cells. The combination of decitabine with GSK343 did not enhance their inhibitory effects and for some combinations were antagonistic. Vorinostat combined with GSK343 enhanced the effects on cell viability in BXD-1425 and EPN1 cell lines, although in general only at higher concentrations.

### HDAC and EZH2 inhibitors were toxic to normal cells

Fetal neural stem cells (fNSC) grown as multicellular spheroids were used to assess toxicity to normal cells. fNSCs were incubated with each drug using the same concentrations as ependymoma cells. The effect on cell viability was measured using an alamar blue assay.

**Figure 4:**
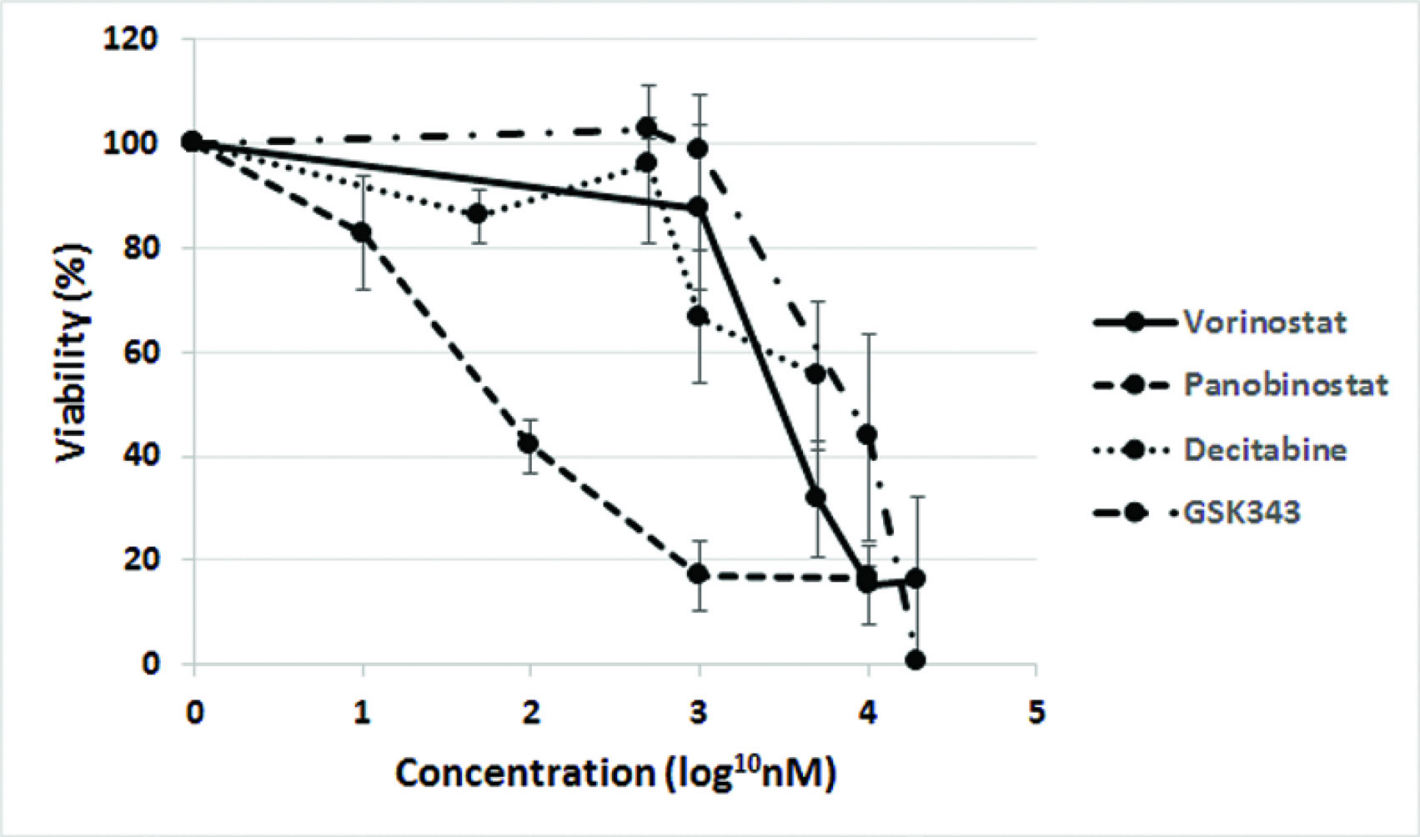
Toxicity of epigenetic agents to fNSCs Epigenetic agents displayed toxicity to fNSCs. Cells were incubated with each drug at the same concentrations and incubation times as ependymoma cells. Viability was measured using an alamar blue assay.

All drugs reduced fNSC viability (Figure 4). The fNSCs were more sensitive to HDAC inhibitors and GSK343 than all ependymoma cells tested. However, they were less sensitive to decitabine than the most sensitive ependymoma cells (BXD-1425EPN, EPN8) (Supplementary Table 2).

## DISCUSSION

Recent research suggests epigenetic modifications play an important role in ependymoma pathology and are therefore a potential therapeutic target, which now needs testing using pre-clinical models. We have shown that agents targeting epigenetic modifications inhibited the growth of cultured ependymoma cells with variable efficiency, suggesting clinical potential in some instances. However, we also demonstrated a lack of similarity between the DNA methylation profiles of the majority of cultured ependymoma cell lines tested and primary ependymoma tumors, with cultured cells appearing closer to cell lines from other brain tumor types. This suggests alterations were induced during culture, resulting in a common DNA methylation profile that overrides the differences normally seen between these tumor types. DNA methylation has been shown to alter during cell culture [22–24] which may explain why the profiles of these cells did not match ependymoma tumors.

These findings highlight the importance of profiling cell lines to measure their validity as a pre-clinical model, and questions the use of 2D cultured cells for pre-clinical testing of epigenetic agents, suggesting more representative models are needed. This might include 3D *in vitro* models. A number of 3D culture systems have been developed, including spheroids, organoids and growth on hydrogels or scaffolds, which allow cells to grow in a system that better reflects tissue architecture and physiological conditions [28, 29]. 3D models of various cancers, including brain tumors, have been shown to have biological profiles closer to the primary tumor than cells cultured in 2D on a flat plastic surface [30–34].

No clear difference was seen in response to epigenetic agents between the one cell line, which retained an ependymoma-like DNA methylation profile (DFKZ-EP1), and those that did not. However, as only one cell line was confidently assigned to an ependymoma subgroup, limited conclusions could be made about how much the alterations in DNA methylation seen during *in vitro* culture altered the response to the agents tested.

The most effective agents against ependymoma cells in this study were the HDAC inhibitors which induced cytotoxicity in the majority of ependymoma cells tested, including DKFZ-EP1, agreeing with previous reports [18, 21]. Milde *et al* also treated the DKFZ-EP1 cell line with vorinostat and panobinostat, identifying IC50 concentrations of 776 nM and 2.9 nM respectively, which were lower than our calculated IC50 concentrations (5 μM and 360 nM). However, Milde *et al* treated the cells when grown as neurospheres instead of a monolayer in serum-supplemented media, which could explain the difference in sensitivity.

Although both vorinostat and panobinostat induced growth inhibition, it was not at clinically achievable concentrations. Reported peak plasma concentrations for vorinostat and panobinostat were 4.5 μM and 40 nM respectively [35, 36], with phase I trials of vorinostat in pediatric solid tumors suggesting this may be lower in pediatric patients (1.4–1.6 μM) [37, 38]. This suggests HDAC inhibitors would not be effective as single agents in patients and agrees with analysis of two HDAC inhibitors in an *in vivo* ependymoma mouse model where low activity was seen [39, 40]. Additionally, although vorinostat was well tolerated in phase I trials of pediatric solid tumors, including ependymoma, limited clinical responses were observed [37, 38]. A combination approach to lower doses needed for an effective response, or a local drug delivery system to enable effective concentrations to reach the tumor while avoiding high systematic doses, could improve the clinical potential of these agents.

Previous research suggested DNA methylation plays an important role in ependymoma [11, 17, 19]. Growth inhibition was only achieved in two cell lines tested, which did not include DKFZ-EP1, questioning the clinical potential of this agent. Mack *et al* previously demonstrated growth inhibition of short-term cultures of cells derived from EPN_PFA tumors [11]. However, profiling was not undertaken to determine if the cells tested retained their original DNA methylation profile.

Where inhibition was achieved IC50 concentrations were within clinical ranges, with reported plasma concentrations up to 5 μM [41]. Decitabine has been shown to cross the blood brain barrier where CSF concentrations were reported at up to 50% of plasma concentrations [42]. Our results demonstrated the effects seen upon treatment with decitabine were cytostatic, suggesting it would need to be used in combination to induce cell death.

The EZH2 inhibitor GSK343 was the least effective of all agents tested. Inhibition was achieved at relatively high concentrations compared to those identified previously in sensitive ependymomas [11]. However, it was closer to IC50 concentrations reported for other cancer types [16, 43]. Pharmacokinetic studies in rat models demonstrated GSK343 displayed high clearance suggesting it is only useful as an *in vitro* experimental tool and not suitable for *in vivo* studies [16]. Alternative EZH2 inhibitors with good pharmacokinetic properties and clinical potential have now been developed, including EPZ-6438, which is currently being investigated in clinical trials with promising preliminary results [13–15]. However, initial pre-clinical testing suggested this drug also displayed low activity in ependymoma [44].

Multiple epigenetic mechanisms can be de-regulated in cancer and studies have shown benefit of targeting multiple mechanisms in combination [45, 46]. However, the combinations tested against ependymoma cells did not show a significant increase in the effects on cell viability.

Epigenetic modifications play a key role in normal brain development [47, 48]. Therefore, caution must be taken when considering targeting these processes for ependymoma therapy. Our analysis suggested the drugs we tested do show toxicity to normal neural stem cells at similar or lower concentrations than those required to inhibit ependymoma cell growth. However, the fetal brain has been shown to have the capacity to respond and repair damage induced by the de-methylating agent 5-azacytidine [49]. Studies investigating toxicity to normal astrocytes and neuronal cells suggest decitabine was not toxic to these cells at concentrations that inhibited growth in sensitive ependymoma cell lines [50, 51]. In contrast, vorinostat and panobinostat have been shown to induce cytotoxicity to normal astrocytes and neurons at similar concentrations to those needed to inhibit ependymoma cell growth [52–54]. However, no neurotoxicity was seen following systematic administration of vorinostat to mice [55], or in a recent human trial of vorinostat in recurrent glioblastoma [56]. Additionally, in clinical trials of pediatric patients, epigenetic drugs have been well tolerated [37, 38, 57, 58]. Further analysis is needed to determine the long-term consequences of any toxicity induced.

In conclusion, we have presented pre-clinical evidence demonstrating epigenetic agents could inhibit the growth of ependymoma cells. However, our results suggest caution is needed in the clinical progression of these agents as inhibitory effects were not always at clinically achievable concentrations and toxicity to normal cells was also seen. Importantly, the alteration of DNA methylation profiles in the majority of cells tested questions the validity of using 2D *in vitro* cultured cell lines as a model and suggests we need more accurate and biologically relevant models for pre-clinical testing to identify successful drug combinations before translation into the clinical setting.

## MATERIALS AND METHODS

### Cell culture

The ependymoma cell line BXD-1425EPN was provided by Dr Xiao-Nan Li, Baylor College of Medicine and has previously been characterized [59]. Cells were derived from a 9-year-old male with a recurrent ST anaplastic ependymoma. The DKFZ-EP1 cell line was provided by Dr T Milde and Dr O Witt, DKFZ and has previously been characterized [21]. Cells were derived from the malignant ascites of an 18-year-old female with a primary grade III classic ST ependymoma. The EPN1 cell line was generated and characterized in house [60]. Cells were derived from a 13-year-old male with a recurrent ST ependymoma. Short term cultures of three PF ependymomas were established as previously described [60]. EPN8 was derived from a 1.5-year-old male with a primary grade III anaplastic PF ependymoma. EPN9 was derived from a 16-month female with a primary grade II PF ependymoma. EPN10 was derived from a 2-year-old female with a PF primary anaplastic ependymoma.

Cells were cultured in standard humidified incubators at 5% CO_2_. BXD-1425EPN and DKFZ-EP1 cells were cultured in DMEM (Invitrogen) supplemented with 10% fetal bovine serum (FBS) (PAA Laboratories) plus antibiotics. EPN1, EPN8, EPN9 and EPN10 were cultured in DMEM supplemented with 15% FBS plus antibiotics.

Doubling times for the cell lines were calculated as BXD-1425EPN; 23 hrs, DKFZ-EP1; 116 hrs, EPN1; 203 hrs, EPN8; 48 hrs, EPN9; 34 hrs, EPN10; 48 hrs. The passage range for the cell lines used were BXD-1425EPN; P66-75, DKFZ-EP1; P10-25, EPN8; p6-15, EPN9; p6-15, EPN10 p6-15.

### Cell line authentication

STR DNA profiling analysis was used for cell line authentication by Eurofins Genomics (Ebersberg, Germany). STR genotypes were generated for two different passages of each line and, where available, the DNA extracted from the tumor tissue the cell line was derived from. Genotypes for all passages of each line matched each other. The genotypes of the primary lines EPN1, EPN8 and EPN10 matched the primary tissue they were derived from, confirming the cultured cells originated from the tumor. No cell line matched any profile in the ATCC and DSMZ databases.

### Determination of C11orf95–rela fusion status

Ependymoma cells were analyzed for the presence of the C11orf95-RELA fusion using western blot of wild-type RELA [20]. Western blots were run as previously described [61]. Membranes were incubated with primary antibodies to RELA (1/10,000, Cell Signalling Technology) and GAPDH (1/50,000, Abcam).

### DNA methylation analysis

DNA was extracted from cells as previously described [19]. Profiling was performed on bisulphite modified DNA using Infinium HumanMethylation450 BeadChip arrays (Illumina) at UCL Genomics (London, UK). All computational analyses were performed in R version 3.4.1 [62]. Raw signal intensities were obtained from IDAT files using the minfi Bioconductor package [63]. Each sample was normalized by performing a background correction (shifting 5% percentile of negative control probes to 0) and a dye-bias correction for both color channels. A correction for the type of material used (FFPE/frozen) was performed by fitting univariate, linear models to the log2-transformed intensity values (removeBatchEffect function, limma package v3.34.5 [64]). Methylated and un-methylated signals were corrected individually. Beta values were calculated from the re-transformed intensities using an offset of 100.

Prior to clustering probes targeting the X and Y chromosomes, probes containing a single nucleotide polymorphism within five base pairs of, and including, the target CpG and probes not mapping uniquely to the human reference genome (hg19) were removed. To perform unsupervised dimension reduction the remaining probes were used to calculate the 1-variance weighted pearson correlation between samples. The resulting matrix was used for input for t-SNE analysis (t-Distributed Stochastic Neighbor Embedding [65], Rtsne package version 0.13 [66]) with the default parameters: theta = 0, pca = F, max_iter = 2500, perplexity = 30.

To predict tumor subgroups a methylation based classifier was used [25] (www.molecularneuropathology.org) which assigns methylation profiles to 91 different tumor subgroups including all ependymoma subgroups previously described [17] and gives a confidences score for the prediction.

Ependymoma cell line methylation profiles have been deposited in NCBI's Gene Expression Omnibus (GEO www.ncbi.nlm.nih.gov/geo) and are accessible through GEO Series accession number GSE111428. Methylation data of ependymomas used for the t-SNE analysis are available in GEO series GSE65362. Methylation data for medulloblastoma and high grade glioma cell lines used for the t-SNE analysis area available in GEO series GSE112067. The medulloblastoma cell lines used were DAOY, UW228-2, ONS-76, D425 MED, D458 MED and D487 MED. The high grade glioma cell lines used were SJ-G2 and SF188. All were cultured under standard conditions prior to DNA extraction and methylation profiling.

### Drugs

All drugs were reconstituted in DMSO. Decitabine (Sigma-Aldrich) at a concentration of 200 mM, vorinostat (Sigma-Aldrich) 250 mM, panobinostat (Selleck Chemicals) 50 mM and GSK343 (Sigma-Aldrich) 28 mM.

### MTT viability assay

Cells were seeded in 96 well plates and incubated overnight. Subsequently, cells were treated with indicated drug or vehicle control (DMSO). For vorinostat, viability was measured after 72 hrs exposure to the drug. For GSK343, drug was replenished after 72 hrs and viability measured 120 hrs after drug treatment started. Cells were treated with decitabine using similar methods to published studies [67–70]. Transient low doses that induce de-methylation but no cytotoxicity, that was unrelated to DNA methylation, were used. Decitabine was replenished 24 and 48 hrs after initial treatment. Viability was measured 144 hrs after drug treatment started.

For combination experiments targeting multiple epigenetic modifications, drugs were used at a fixed ratio based on single agent IC50 concentrations. Cells were treated with 0.125, 0.25, 0.5, 1, 2 and 4 times the IC50 concentration. For combinations involving decitabine, cells were pre-treated for 48 hrs with decitabine before addition of the second drug after which cells were incubated for a further 96 hrs. Cells were incubated with vorinostat in combination with GSK343 for 72 hrs.

Cell viability was measured using the Cell proliferation kit I (MTT) (Roche), following manufacturer's instructions. Each condition was performed in triplicate, in three independent repeats. The percentage of viable cells relative to vehicle control was calculated based on averaged triplicates for each condition. IC50 concentrations were estimated from dose response curves.

### LDH assay

Cells were seeded in 96 well plates and incubated overnight. Subsequently, cells were treated with indicated drug or vehicle control. Cells were incubated for 72 hrs with vorinostat, 120 hrs with GSK343 and 144 hrs with decitabine before analysis. GSK343 was replenished after 72 hrs and decitabine after 24 and 48 hrs. Cell death was measured using the Cytotoxicity Detection Kit^PLUS^ (LDH) (Roche), following manufacturer's instructions. Each condition was performed in triplicate, in three independent repeats.

### Toxicity analysis

Fetal neural stem cells (fNSC) were derived and cultured as previously described [71]. 10,000 cells per well were seeded into Ultra low attachment 96-well round bottom plates and incubated for 48 hrs, allowing time for formation of a single uniformly sized spheroid. Subsequently, indicated drug or vehicle control was added and incubated for indicated time period. Decitabine was replenished after 24 and 48 hrs. Cell viability was measured using an alamar blue assay (ThermoFisher Scientific), following manufacturer's instructions. Each condition was performed in triplicate, in three independent repeats.

## SUPPLEMENTARY MATERIALS FIGURES AND TABLES



## Abbreviations

H3K27: lysine 27 of histone H3
PRC2: polycomb repressor complex 2
EZH2: enhancer of zeste homolog 2
PF: posterior fossa
ST: supratentorial
fNSC: fetal neural stem cell
