# Supplementary material for: Limitations of current in vitro models for testing the clinical potential of epigenetic inhibitors for treatment of pediatric ependymoma
Source: Oncotarget. 2018 Nov 23;9(92):36530–41. doi: 10.18632/oncotarget.26370 (PMC6284855; doi:10.18632/oncotarget.26370)
Supplement: Supplementary file 1 [file oncotarget-09-36530-s001.pdf]

Limitations of current *in vitro* models for testing the clinical potential of epigenetic inhibitors for treatment of pediatric ependymoma

SUPPLEMENTARY MATERIALS

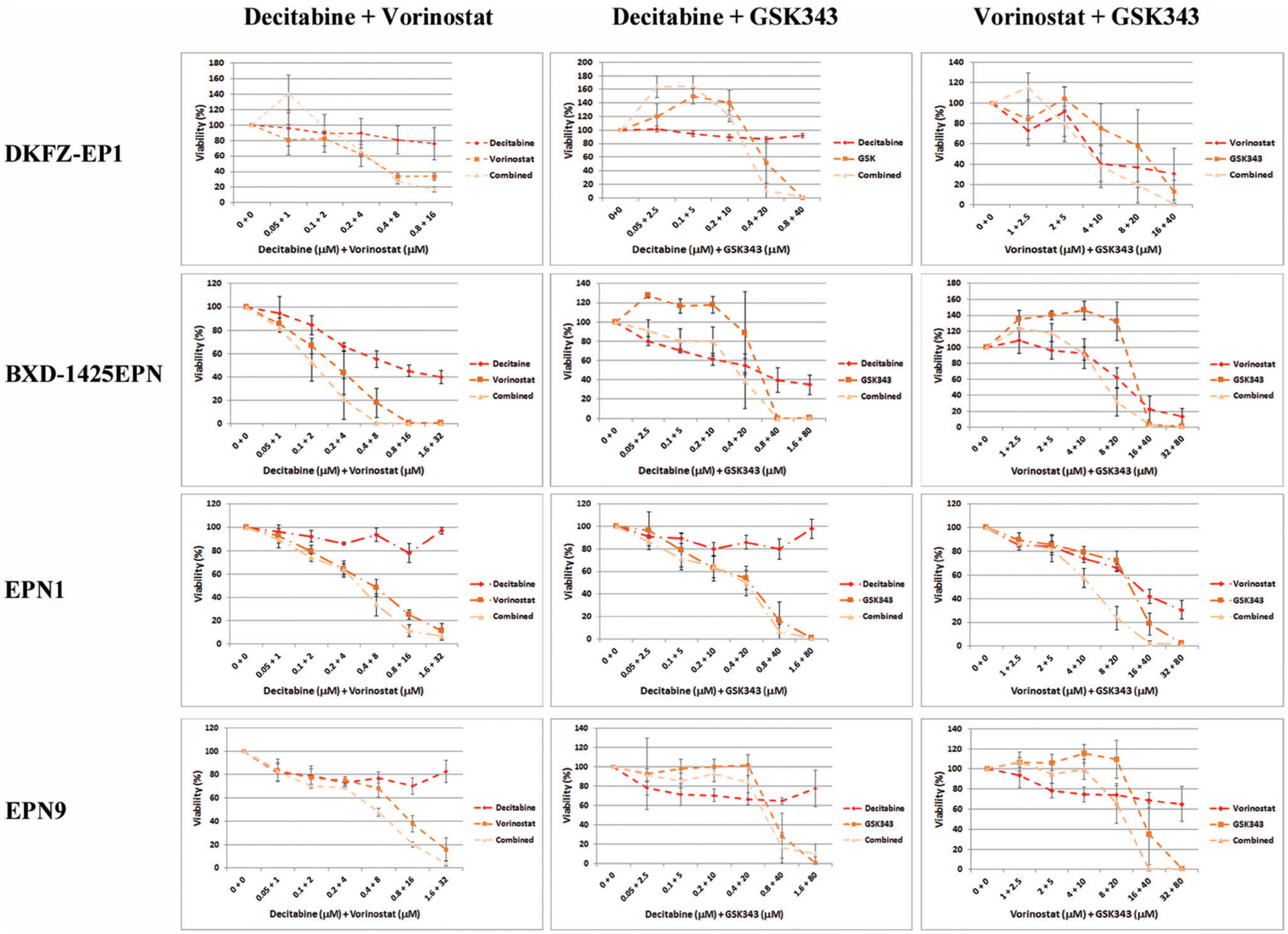

Supplementary Figure 1: Effect of epigenetic agent combinations on ependymoma cell line viability. Ependymoma cells were treated with combinations of epigenetic agents after which cell viability was measured using a MTT assay. Ependymoma cells were pre-treated with decitabine for 48 hrs before addition of vorinostat or GSK343 after which cells were incubated for a further 96 hours. The combination of decitabine and vorinostat enhanced the effect on cell viability in some cell lines, but only at higher concentrations. No enhancement was seen for combinations of decitabine and GSK343. Ependymoma cells were incubated with vorinostat in combination with GSK343 for 72 hrs. The combination enhanced the effect on cell viability in some cell lines but generally only at higher concentrations.

Supplementary Table 1: IC50 concentrations (µM) for ependymoma cells treated with agents targeting epigenetic modifications

	Vorinostart	Panobinostat	Decitabine	GSK343
BXD-1425EPN	8	0.09	0.4	16
DKFZ-EP1	5	0.36	-	20
EPN1	20	0.23	-	-
EPN8	10.5	0.08	0.05	20
EPN9	-	0.47	-	-
EPN10	-	0.51	-	-

If no value is displayed an IC50 was not reached.

Supplementary Table 2: IC50 concentrations (µM) for fNSC treated with agents targeting epigenetic modifications

	IC50 (µM)
Vorinostat	4
Panobibostat	0.08
Decitabine	-
GSK343	9

If no value is displayed an IC50 was not reached.
